# Supplementary material for: MetaRibo-Seq measures translation in microbiomes
Source: Nat Commun. 2020 Jun 29;11:3268. doi: 10.1038/s41467-020-17081-z (PMC7324362; doi:10.1038/s41467-020-17081-z)
Supplement: Supplementary file 10 — Supplementary Data 7 [file 41467_2020_17081_MOESM10_ESM.zip › File2/Confidence_High_Taxonomy/116821_out.krona.html]

Javascript must be enabled to view this page.

members
magnitude
magnitudeUnassigned
count
unassigned
taxon
rank

116821\_out

4

2
4
superkingdom

1239
4
phylum

4
186801
class

186802
4
order

186804
4
family

genus
1913599
4


SRS023595\_contig\_number\_72533SRS047634\_contig\_number\_14862SRS063603\_contig\_number\_contig-100\_674.192511SRS104711\_contig\_number\_13315
4
796937
species
